# A prognostic model for patients with primary extramedullary multiple myeloma

**DOI:** 10.3389/fcell.2022.1021587

**Published:** 2022-11-25

**Authors:** Limei Zhang, Shuzhao Chen, Weida Wang, Yun Wang, Yang Liang

**Affiliations:** ^1^ Sun Yat-sen University Cancer Center, State Key Laboratory of Oncology in South China, Collaborative Innovation Center for Cancer Medicine, Guangzhou, China; ^2^ Department of Hematologic Oncology, Sun Yat-sen University Cancer Center, State Key Laboratory of Oncology in South China, Guangzhou, China

**Keywords:** extramedullary multiple myeloma, prognostic factors, overall survival, prognostic nomogram, progression-free survival

## Abstract

**Background:** Extramedullary disease is a manifestation of multiple myeloma, the prognosis of which remains poor even in the era of novel drugs. Therefore, we aimed to develop a predictive model for patients with primary extramedullary multiple myeloma (EMM).

**Methods:** Clinical and laboratory data of patients diagnosed with primary EMM between July 2007 and July 2021 were collected and analyzed. Univariate and least absolute shrinkage and selection operation Cox regression analyses (LASSO) were used to select prognostic factors for overall survival (OS) to establish a nomogram prognostic model. The performance of the model was evaluated using concordance index which was internally validated by bootstraps with 1,000 resample, area under the curve (AUCs), and calibration curves.

**Results:** 217 patients were included in this retrospective study. Patients with EMM had a higher rate of belonging to the male sex, age >50 years, advanced Durie–Salmon stage III, hypercalcemia, and low hemoglobin level. Compared with patients with bone-related extramedullary disease, those with extraosseous-related extramedullary disease had a higher frequency of advanced Durie–Salmon stage III, lower rate of hypercalcemia, and elevated prothrombin time. The OS and progression-free survival (PFS) of patients with bone-related extramedullary disease were significantly higher than those of patients with extraosseous-related extramedullary disease. After the univariate and LASSO analyses, six prognostic factors, including performance status, number of extramedullary involved sites, β2-microglobulin, lactate dehydrogenase, monocyte–lymphocyte ratio, and prothrombin time, were integrated to establish a nomogram. The model showed robust discrimination with a concordance index (C-index) of 0.775 (95% confidence interval [CI], 0.713–0.836), internally validated with the corrected C-index of 0.756, and excellent performance in time-dependent AUCs compared with other staging systems. The AUCs for 1-, 3-, and 5-year OS were 0.814, 0.744, and 0.832, respectively. The calibration curves exhibited good consistency between the observed and nomogram-predicted OS. The 5-year OS of patients in the high-risk group (23.3%; 95% CI, 13.9%–39.3%) was much worse than that in the low-risk group (73.0%; 95% CI, 62.5%–85.4%; *p* < 0.001).

**Conclusion:** The nomogram predictive model based on six clinical variables showed good prognostic performance and could better predict individual survival in patients with EMM.

## Introduction

Multiple myeloma (MM) is the second most prevalent hematologic malignancy in high-income countries. It is characterized by an abnormal accumulation of clonal plasma cells in the bone marrow, resulting in hypercalcemia, poor renal function, anemia, and bone destruction (Cowan et al., 2018; van de Donk et al., 2021). MM cells are always confined within the bone marrow in classic MM. However, as a special manifestation of MM, extramedullary multiple myeloma (EMM) results from MM cells escaping from the bone marrow and infiltrating many organs, including the skin, kidney, breast, lung, or even the central nervous system, occurring at the time of diagnosis (primary EMM) or at the time of replication (secondary EMM) (Bladé et al., 2011; Weinstock and Ghobrial, 2013; Gagelmann et al., 2018). The definition of EMM has not yet reached a consensus. Several studies have defined EMM as soft tissue masses in extraosseous locations due to hematogenous spread, and their presence is not related to the bone (Usmani et al., 2012; Weinstock and Ghobrial, 2013; Touzeau and Moreau, 2016; Bladé et al., 2022). However, according to some studies, EMM can be divided into two types. The first type is bone-related extramedullary disease (EM-B), in which the MM directly extends *via* disruption of cortical bones into adjacent soft tissues. The second type is extraosseous-related extramedullary disease (EM-E) resulting from hematogenous spread, in which the MM is located in organs and soft tissues that do not adjoin bones (Varettoni et al., 2010; Usmani et al., 2012; Weinstock and Ghobrial, 2013; Touzeau and Moreau, 2016). The survival outcome of EM-E was reported to be worse than that of EM-B (Pour et al., 2014; Batsukh et al., 2017).

With the development of new drugs, including proteasome inhibitors, immunomodulatory agents, monoclonal antibodies, and autologous stem cell transplantation, MM survival has achieved encouraging outcomes (Kumar et al., 2017b). However, compared with MM without extramedullary disease, the prognosis of EMM remains poor even in the era of novel drugs (Usmani et al., 2012; Bhutani et al., 2020). The molecular mechanism of EMM remains unclear. When the plasma cells escape from the cellular microenvironment, plasma cell leukemia or spread to soft tissues in the form of plasmacytomas may happen. The escape is driven by pathophysiological alterations. The possible mechanism of MM extramedullary spread may include decreased expression of adhesion molecules, downregulation of chemokine receptors and tetraspanins expression, increased heparanase-1 expression (Bladé et al., 2011). Therefore, the standard treatment strategy of EMM has not been fully estalished yet.

As the low prevalence of EMM, there were limited data on the baseline characteristics of EMM. According to the different clinical characteristics and prognosis of EMM, the current staging systems for MM are not accurate and specific enough for the survival prediction of EMM. Up to now, there is no prognostic model designed for patients with EMM. Therefore, the present study aimed to analyze the clinical characteristics, prognostic factors and survival status of patients with primary EMM and developed a nomogram prognostic model based on prognostic factors for EMM survival predictive optimization. As a result, the treatment optimization could be appled to patients with EMM to improve their prognosis.

## Patients and methods

### Patient selection

We retrospectively collected and analyzed the clinical and laboratory data of patients diagnosed with primary EMM between July 2007 and July 2021 at the Sun Yat-sen University Cancer Center. The inclusion criteria of patients included in our study were as follows:1) age≥18 years; 2) newly diagnosed multiple myeloma based on the World Health Organization (WHO) or the International Myeloma Working Group (IMWG) diagnostic criteria; 3) presention with extramedually lesions at the time of diagnosis and not after treatment; 4) all patients should receive chemotherapy (proteasome based regimen or immunomodulatory based regimen or other types of chemotherapy) not just only palliative care; 5) baseline clinical and laboratory data could be obtained before treatment. Patients presenting with evidence (pathological or radiological) of neoplastic plasma cells in the soft tissues adjacent to axial skeleton were deemed as have EM-B, Presention in organs and soft tissues that do not adjoin bones were regarded as having EM-E. Cases with both EM-B and EM-E were included in the EM-E group. Patients diagnosed of plasma cell leukemia or solitary plasmacytoma were excluded from our study. The following data were recorded: age at diagnosis, sex, number of extramedullary sites, Durie–Salmon (DS) stage, International Staging System (ISS), transplant, radiotherapy, types of chemotherapy, C4 complement, lactate dehydrogenase (LDH), β2-microglobulin (β2-MG), creatinine, calcium, platelets, hemoglobin (Hb), monocyte–lymphocyte ratio (MLR), neutrophil–lymphocyte ratio (NLR), and prothrombin time. Patients lacking one of the above covariates were excluded from this study.

### Follow-up

Overall survival (OS) was defined as the time interval from the date of diagnosis to the date of death caused by any cause or the time of the last follow-up. OS was our primary end point. Progression-free survival (PFS) was calculated from the date of diagnosis to the date of disease progression, death from any cause, or the last follow-up. PFS was our second end point.

### Statistical analysis

The cut-off value of the continuous variates was determined using the maximal log-rank statistics, which divided the patients with EMM into higher than the cut-off value group and lower than the cut-off value group. Differences in parameters between the two groups (EM-B and EM-E) were compared using the chi-square test. Univariate Cox regression analysis and the least absolute shrinkage and selection operation (LASSO) Cox regression model were used to screen for prognostic factors for model construction. The variates with *p*-value less than 0.05 in univariate Cox regression analysis were analyzed in LASSO Cox analysis. The candidate variables were further filtered using the LASSO Cox regression model with the criteria of 1-se. The selected prognostic factors were then integrated into the prognostic model with the coefficients identified by the nomogram algorithm. The prognostic accuracy of the model was estimated using the concordance index and area under the curve (AUC) at different times. Performance evaluation of the predictive nomogram model also included a comparison between other staging systems of MM by using time-dependent receiver operating characteristic curves. Calibration curves were used to compare the consistency between the real observed survival and nomogram-predicted survival. For internal validation, a bootstrap with 1,000 resamples was used. Each patient with EMM had its own risk score. The median risk score was used to divide the patients into high- and low-risk groups. We performed the Kaplan-Meier method to estimate survival and compare the differences between survival curves using the log-rank test. A two-sided *p*-value less than 0.05 was deemed statistically significant. All statistical analyses were conducted using SPSS 22 and R 4.0.3.

## Results

### Baseline characteristics

**FIGURE 1:**
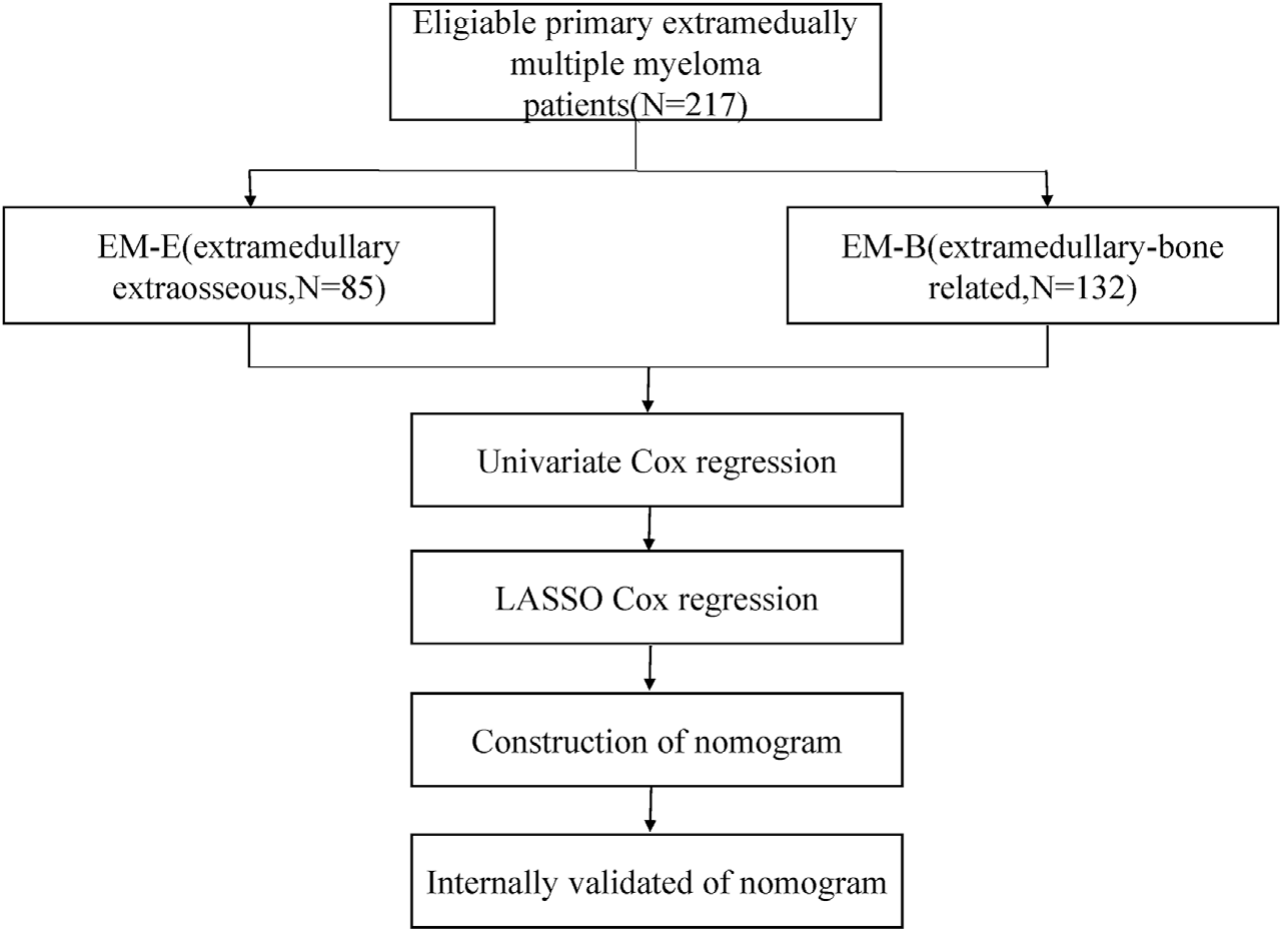
Flow chart of this study.

**FIGURE 2:**
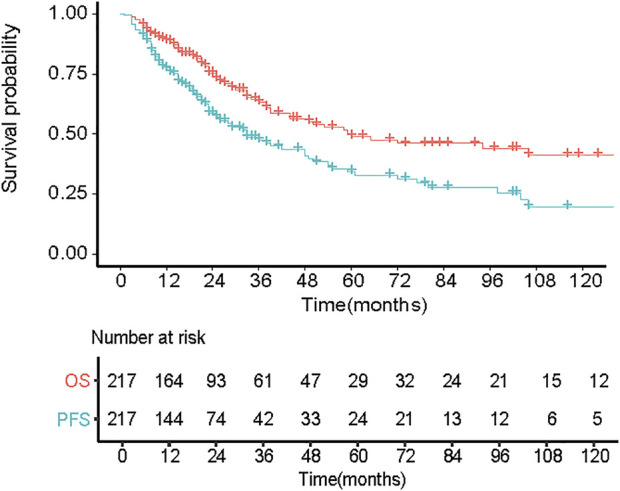
Overall survival (OS) and progression free disease (PFS) of 217 patients with primary EMM.

The flow chart of our study can be shown in Figure 1A total of 217 eligible patients with primary EMM between July 2007 and July 2021 at the Sun Yat-sen University Cancer Center were included in this retrospective study. The baseline characteristics of the patients are presented in Table 1. The median age of the patients was 60 years, and 85.3% were over 50 years old. Of the patients, 62.2% were male. Most patients showed a good performance status (Eastern Cooperative Oncology Group [ECOG] score <2). In addition, patients with EMM had a higher rate of advanced DS stage III, hypercalcemia, and low Hb levels. One extramedullary site was involved in 66.8% of the patients. Among the 217 patients, 85 (39.2%) presented with EM-B and 132 (60.8%) had EM-E. Compared with patients with EM-B, those with EM-E had a higher frequency of advanced DS stage III, lower rate of hypercalcemia, and elevated prothrombin time (all *p* < 0.05). About 65% patients received proteasome inhibitors or immunomodulatory drugs or commbination. The distribution of the sites involved in patients with EM-E is shown in Table 2. The soft tissues (muscle/skin), kidney, and lymph nodes were the top three extramedullary sites in patients with EM-E, and the total proportions of the above sites reached 50% in patients with EM-E (Table 2). The OS and PFS of all patients are shown in Figure 2. The 3-year OS and PFS rates were 63.3% (95% confidence interval [CI], 55.6%–72.1%) and 47.5% (95% CI, 39.8%–56.7%), respectively. The 5-year OS and PFS rates were 49% (95% CI, 40.3%–59.6%) and 34.3% (95% CI, 26.1%–44.9%), respectively.

**TABLE 1:** Clinical characteristics of patients with extramedullary multiple myeloma.

Characteristics	Total (N = 217) no. (%)	EM-E (N = 85) no. (%)	EM-B(N = 132) no. (%)	*p*
Age, median, range (years)	60 (23–94)	58 (25–94)	61 (23–81)	0.334
<50	32 (14.7)	15 (17.6)	17 (12.9)	
≥50	185 (85.3)	70 (82.4)	115 (77.1)	
Sex				0.801
Male	135 (62.2)	52 (61.2)	83 (62.9)	
Female	82 (37.8)	33 (38.8)	49 (37.1)	
Number of involved sites				0.214
1	145 (66.8)	61 (71.8)	84 (63.6)	
≥2	72 (33.2)	24 (28.2)	61 (36.4)	
DS stage				**0.021**
I	20 (9.2)	9 (6.8)	11 (12.9)	
II	30 (13.8)	13 (9.8)	17 (20)	
III	167 (77.0)	110 (83.3)	57 (67.1)	
ISS stage				0.433
I	73 (33.6)	29 (34.1)	44 (33.3)	
II	45 (20.7)	21 (24.7)	24 (18.2)	
III	99 (45.6)	35 (41.2)	64 (48.5)	
R-ISS stage				0.872
I	25 (11.5)	9 (10.6)	16 (12.1)	
II	91 (41.9)	38 (44.7)	53 (40.2)	
III	29 (13.4)	12 (14.1)	17 (12.9)	
Unknown	72 (33.2)	26 (30.6)	46 (34.8)	
Transplant				0.648
Yes	23 (10.6)	8 (9.4)	15 (11.4)	
No	194 (89.4)	77 (90.6)	117 (88.6)	
Radiotherapy				0.488
Yes	17 (7.8)	8 (9.4)	9 (6.8)	
No	200 (92.2)	77 (90.6)	123 (93.2)	
Treatment				0.374
PIs	37 (17.1)	15 (17.6)	22 (16.7)	
IMiDs	57 (26.3)	17 (20.0)	40 (30.3)	
IMiDs-PIs	46 (21.2)	21 (24.7)	25 (18.9)	
Other	77 (35.5)	32 (37.6)	45 (34.1)	
Cytogenetics				0.772
High risk	38 (17.5)	13 (15.3)	25 (18.9)	
No risk	68 (31.3)	28 (32.9)	40 (30.3)	
Missing	111 (51.2)	44 (51.8)	67 (50.8)	
ECOG				0.102
0–1	191 (88)	71 (83.5)	120 (90.9)	
≥2	26 (12)	14 (16.5)	12 (9.1)	
C4 (g/L)				0.177
<0.45	194 (89.4)	73 (85.9)	121 (91.7)	
≥0.45	23 (10.6)	12 (14.1)	11 (8.3)	
Albumin (g/L)				0.142
<40	135 (62.2)	58 (68.2)	77 (58.3)	
≥40	82 (37.8)	27 (31.8)	55 (41.7)	
LDH (U/L)				0.256
<250	186 (85.7)	70 (82.4)	116 (87.9)	
≥250	31 (14.3)	15 (17.6)	16 (12.1)	
β2-MG (mg/L)				0.213
<3.5	111 (51.2)	39 (45.9)	72 (54.5)	
≥3.5	106 (48.8)	46 (54.1)	60 (45.5)	
CRE (umol/L)				0.326
≥176	18 (8.3)	123 (93.2)	76 (89.4)	
<176	199 (91.7)	9 (6.8)	9 (10.6)	
Ca (mmol/L)				**0.042**
<2.2	67 (30.9)	33 (38.8)	34 (25.8)	
≥2.2	150 (69.1)	52 (61.2)	98 (74.2)	
PLT (10E9/L)				0.120
<150	40 (18.4)	20 (23.5)	20 (15.2)	
≥150	177 (81.6)	65 (76.5)	112 (84.8)	
Hb(g/L)				0.052
<125	144 (66.4)	63 (74.1)	81 (61.4)	
≥125	73 (33.6)	22 (25.9)	51 (38.6)	
MLR				0.053
<0.32	154 (71.0)	54 (63.5)	100 (75.8)	
≥0.32	63 (29.0)	31 (36.5)	32 (24.2)	
NLR				0.524
<4.2	195 (89.9)	120 (90.9)	75 (88.2)	
≥4.2	22 (10.1)	12 (9.1)	10 (11.8)	
PT(s)				**0.038**
<12.7	169 (77.9)	109 (82.6)	60 (70.6)	
≥12.7	48 (22.1)	23 (17.4)	25 (29.4)	

Abbreviations: DS, Durie-Salmon stage; ISS, international staging system; PIs, proteasome inhibitors; IMiDs, immunomodulatory drugs; ECOG, eastern cooperative oncology group; C4, complement; LDH, lactate dehydrogenase; Ca, Calcium; β2-MG, β2-microglobulin; CRE, creatinine; Ca, calcium; PLT, platelet; Hb, hemoglobin; MLR, monocyte-lymphocyte ratio; NLR, neutrophil-lymphocyte ratio; PT, prothrombin time. The bold p values meaned the variables between EM-B and EM-E groups were different with statistical significance (p <0.05).

**TABLE 2:** Distributions of involved sites in patients with EM-E.

Group	Extramedullary sites	No. (%)
	Soft tissues (muscle/skin)	23 (27.1)
	Kidney	16 (18.8)
	Lymph nodes	12 (14.1)
	Pleural	7 (8.2)
	Lung	5 (5.9)
EM-E (N = 85)	Breast	5 (5.9)
	Chest wall	5 (5.9)
	Oropharynx	4 (4.7)
	Stomach	3 (3.5)
	Testis	2 (2.4)
	Liver	2 (2.4)
	Thyroid	1 (1.1)

### Prognostic factors and model construction

**FIGURE 3:**
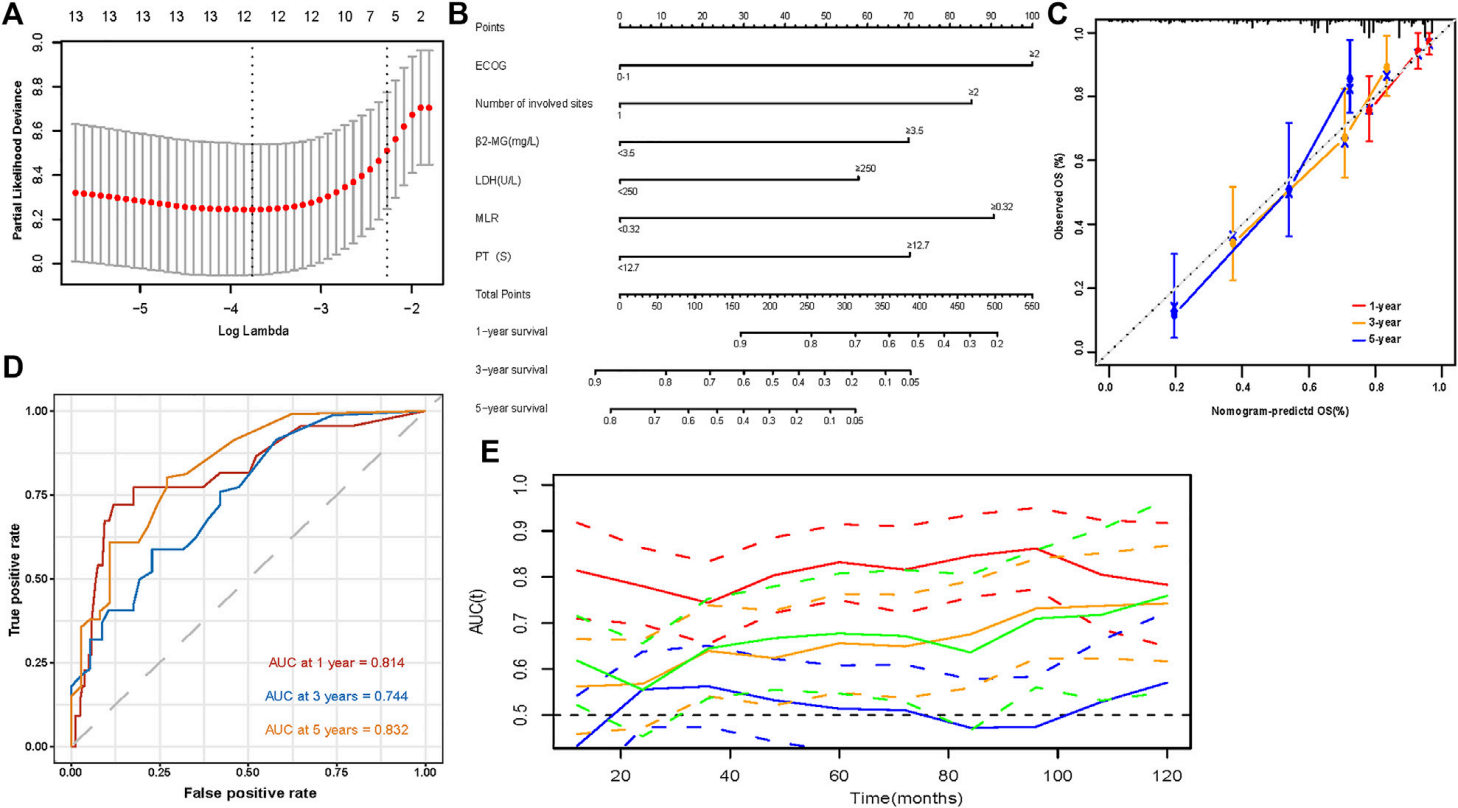
Development and evaluation of a predictive model **(A)** 1000 bootstrap replicates by Lasso Cox regression analysis for variable selection; **(B)** The nomogram based on data from 217 patients with primary EMM to predict individual prognosis; **(C)** Calibration curves for predicting OS at 1-, 3-, and 5-year; **(D)** Sensitivity and specificity at 1-, 3-, and 5-year of the predictive nomogram model were assessed in 217 patients by time-dependent ROC analysis; **(E)** Time-dependent receiver operating characteristic (ROC) curves (Nomogram [red], R-ISS [green], ISS [orange], DS [blue]).

We performed a univariate analysis of OS in patients. Radiotherapy and transplant status were not included in candidate factor screening. This was because therapy information was not a prognostic variable that could be acquired at the initial diagnosis. In addition, we excluded the DS, ISS, and R-ISS staging systems in univariate analysis, causing the above staging systems to have a collinearity relationship with other variates. We wanted to build an independent prognostic model beyond the existing staging system. Univariate analysis of OS in patients with EMD is shown in Table 3. Except for sex and creatinine and calcium levels, the remaining variables were statistically significant prognostic factors in the initial univariate Cox analysis. We then used the candidate prognostic variates in the LASSO Cox regression analysis to determine the prognostic factors. Finally, six clinical prognostic factors, including ECOG, number of extramedullary sites involved, β2-MG, LDH, MLR, and prothrombin time, were identified (Figure 3A). All six clinical parameters were integrated to develop a nomogram prognostic model (Figure 3B). The formula of the risk score depending on the nomogram was calculated as follows: 0.5370 × ECOG (≥2) + 0.1394 × number of extramedullary involved sites (≥2) + 0.1314 × β2-MG (≥3.5 mg/L) + 0.0392 × LDH (≥250 U/L) + 0.3042 × MLR (≥0.32) + 0.1407 × PT (≥12.7 s).

**TABLE 3:** Univariate analysis of OS in patients with EMD.

Variates	HR	95% CI	*p*
Age (≥50 vs. < 50)years	2.166	1.035–4.533	0.040*
Sex (Female vs. Male)	0.986	0.611–1.589	0.953
EMM(EM-E vs. EM-B)	1.731	1.075–2.787	0.024*
Number of involved sites (≥2 vs. 1)	2.277	1.426–3.636	0.001*
ECOG (≥2 vs. 0–1)	3.879	2.243–6.710	< 0.001*
C4 (≥0.45 vs. < 0.45) g/L	1.953	1.070–3.563	0.029*
Albumin (≥40 vs. < 40) (g/L)	0.447	0.261–0.765	0.003*
LDH (≥250 vs. < 250) (U/L)	2.981	1.687–5.269	< 0.001*
β2-MG (≥3.5 vs. < 3.5) (mg/L)	2.423	1.495–3.927	< 0.001*
CRE(≥176 vs. < 176) umol/L	1.032	0.446–2.385	0.942
Ca (≥2.2 vs. < 2.2) mmol/L	0.716	0.445–1.153	0.169
PLT (≥150 vs. < 150) 10E9/L	0.441	0.264–0.737	0.002*
Hb(≥125 vs .< 125) g/L	0.416	0.238–0.728	0.002*
MLR (≥0.32 vs. < 0.32)	2.642	1.653–4.223	< 0.001*
NLR(≥4.2 vs. < 4.2)	2.016	1.059–3.840	0.033*
PT (≥12.7 vs. < 12.7)s	2.479	1.518–4.048	< 0.001*

### Evaluation and validtion of the nomogram predictive model

**FIGURE 4:**
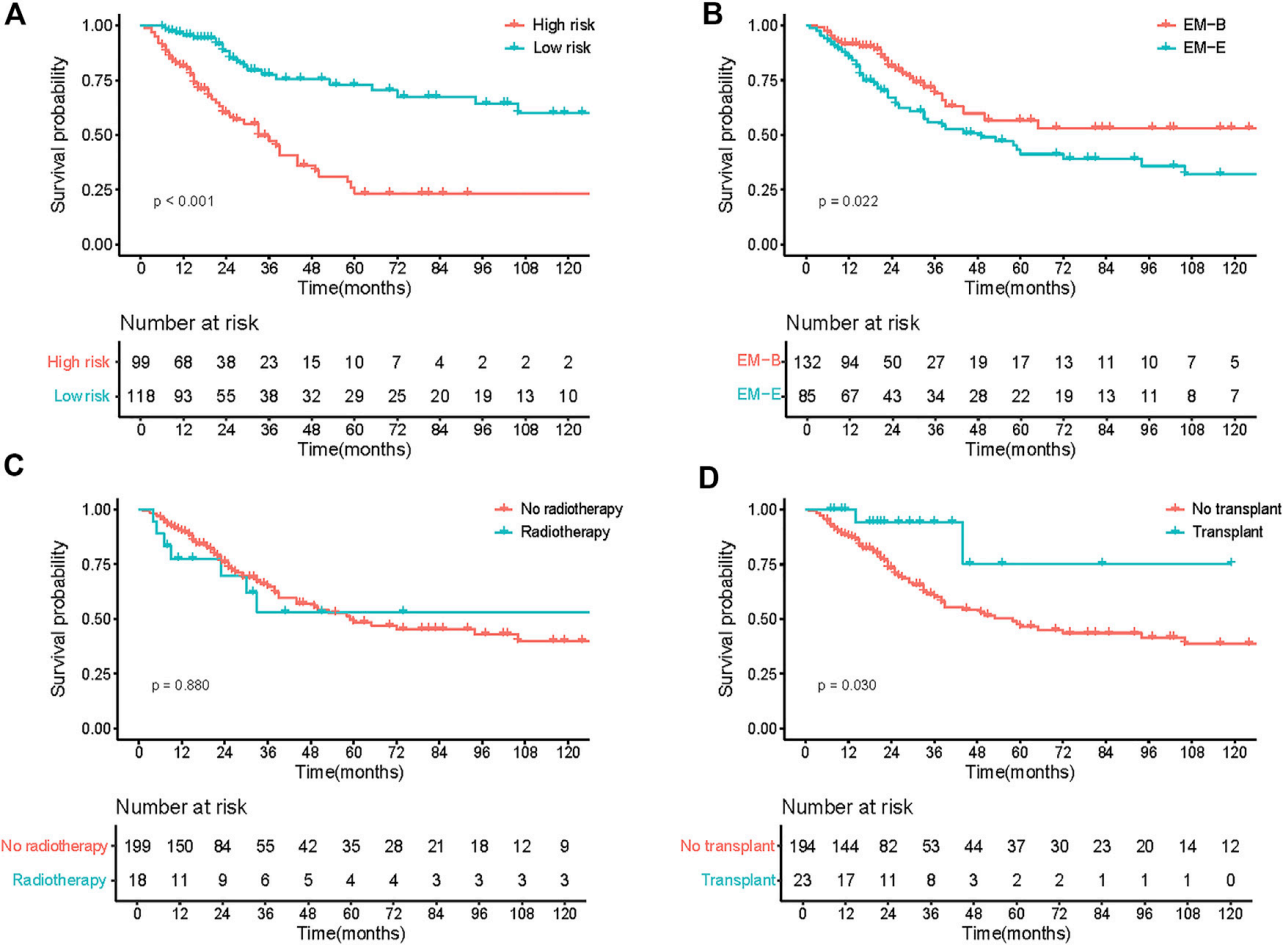
OS of patients according to different stratification. **(A)** Survival curves of risk stratification based on our model (High risk vs Low risk). **(B)** Survival curves of different groups (EM-B vs EM-E). **(C)** Survival curves of different treatment (No radiotherapy vs Radiotherapy). **(D)** Survival curves of different treatment (No transplant vs Transplant).

**FIGURE 5:**
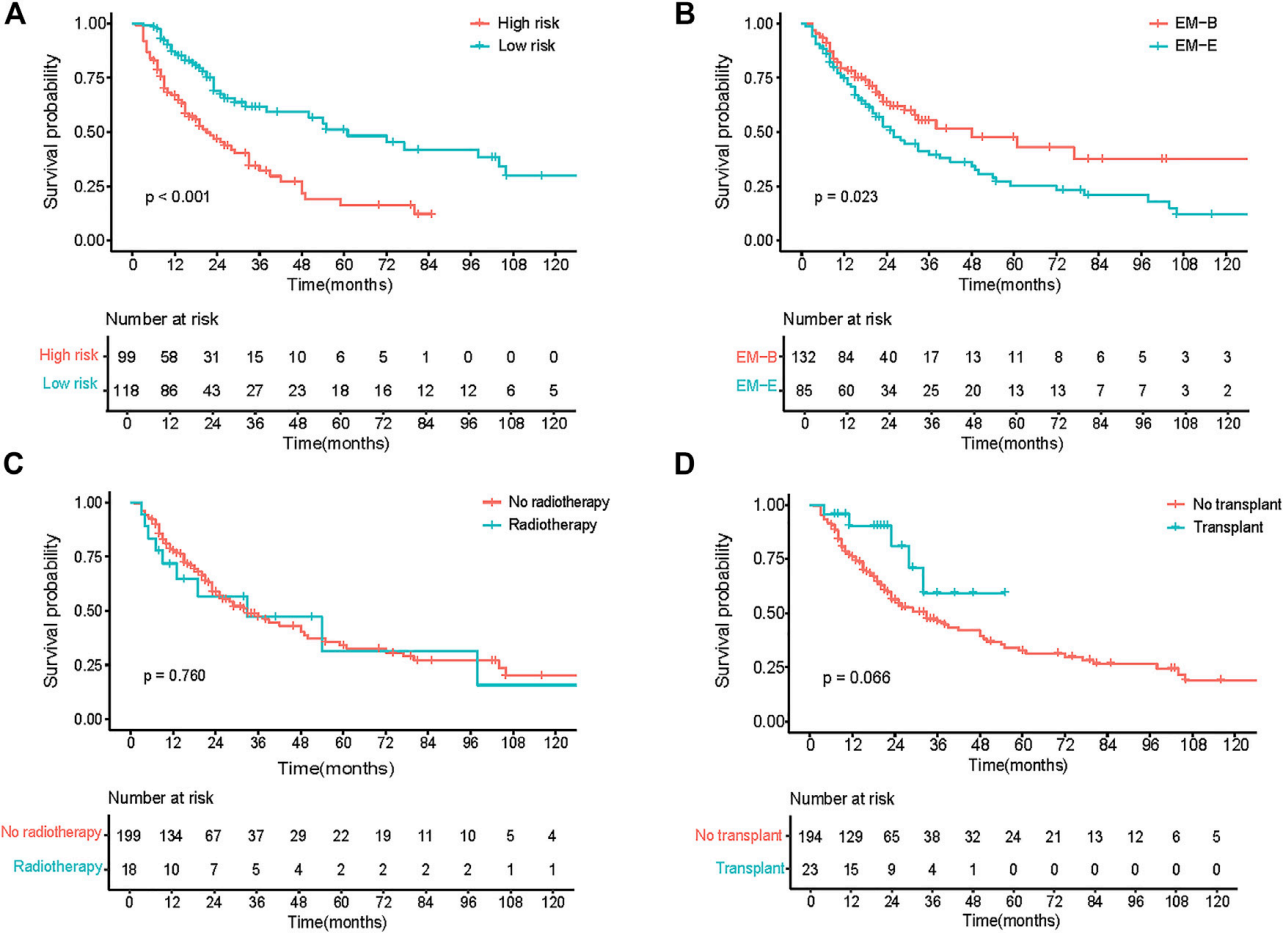
PFS of patients according to different stratification. **(A)** Survival curves of risk stratification based on our model (High risk vs Low risk). **(B)** Survival curves of different groups (EM-B vs EM-E). **(C)** Survival curves of different treatment (No radiotherapy vs Radiotherapy). **(D)** Survival curves of different treatment (No transplant vs Transplant).

The model showed good discrimination, with a concordance index of 0.775 (95% CI, 0.713–0.836). The model was internally validated by bootstraps with 1,000 resample with the corrected C-index of 0.756 and the corrected *R* ^2^ of 0.182. The calibration curves at 1-, 3-, and 5-year also exhibited excellent consistency between the real observed survival and nomogram-predicted survival (Figure 3C). The AUCs for the 1-, 3-, and 5-year OS were 0.814, 0.744, and 0.832, respectively (Figure 3D). Compared with other staging systems for MM, the established nomogram model possessed a higher AUC (Figure 3E). Based on the built model, the median risk score was used to divide patients into high- and low-risk groups for both OS and PFS. The OS of patients with primary EMM at a low risk was significantly better than that of patients at a high risk (Figure 4A, *p* < 0.001). The 5-year OS of patients in the high-risk group was 23.3% (95% CI, 13.9%–39.3%), which was worse than that in the low-risk group (73.0%, 95% CI, 62.5%–85.4%, *p* < 0.001). Patients with EM-B showed a significantly better OS than those with EM-E (Figure 4B, *p* = 0.022). Radiotherapy did not provide a survival advantage in patients with EMM (Figure 4C, *p* = 0.880). However, transplantation resulted in significant OS benefits in patients (Figure 4D, *p* = 0.030). Regarding PFS, patients at a high risk still exhibited worse survival than those at a low risk (Figure 5A, *p* < 0.001). The 3-year PFS of patients in the high-risk group was 32.1% (95% CI, 22.6%–45.8%), which was much worse than that in the low-risk group (61.7%, 95% CI, 51.5%–74.0%, *p* < 0.001). Patients with EM-B also showed better PFS than those with EM-E (Figure 5B, *p* = 0.023). Neither radiotherapy nor transplantation could improve PFS in patients with EMM (Figures 5C,D, *p* > 0.05).

## Discussion

In this study, we analyzed the characteristics of patients with primary EMM and explored the independent prognostic factors to build a prognostic model. We found that the following six factors had a strong impact on OS: ECOG, number of extramedullary involved sites, β2-MG, LDH, MLR, and PT. Based on the above variables, we developed a prognostic nomogram model with robust survival predictive performance, including excellent discriminative ability and satisfactory predictive consistency between observed survival and nomogram-predicted survival probabilities. Our study is the first to establish a nomogram model to predict individual survival.

The 3- and 5-year OS rates of all patients were 63.3% and 49%, respectively, which were similar to those reported previously (68.4% and 53.8%, respectively) (Li et al., 2021). In a retrospective study enrolling 226 patients with EMM between 2010 and 2017, among patients with primary EMM, the median OS was 46.5 months for EM-E and not reached for EM-B (Beksac et al., 2020). In our study, the median OS was 49 months, which was not reached for EM-B, similar to that reported in a previous study. In our study, both OS and PFS in patients with EM-B were significantly better than those in patients with EM-E. This suggests consistency with previous studies that EM-E has much worse biological behavior than EM-B (Pour et al., 2014; Touzeau and Moreau, 2016).

EM involvement always suggests poor prognosis in MM, with high mortality (Usmani et al., 2012). Various factors can influence the outcome of patients with EMM. The following factors can lead to worse survival: 1) secondary EMM; 2) bone-independent EMM; 3) multiple organ involvement; 4) central nervous system involvement; 5) no transplant; 6) incomplete response post-transplant; 7) high β2-MG; 8) anemia; 9) thrombocytopenia; 10) elevated serum LDH; and 11) cytogenetic abnormalities (Gozzetti et al., 2012; Kumar et al., 2017a; Gagelmann et al., 2018; Beksac et al., 2020). In our study, EM-E, multiple extramedullary involved sites (≥2), high β2-MG, low Hb, low platelet count, no transplant, and elevated serum LDH were correlated with poor OS, which was consistent with the above prognostic factors. Several studies have suggested that early transplantation can benefit patients with EMM (Bianchi et al., 2014; Li et al., 2014; Lee et al., 2015), which is opposite to the finding in other studies (Pérez-Simón et al., 2006; Minnema et al., 2008; Varettoni et al., 2010). Besides, many studies have shown that performance status is highly associated with survival in patients with MM (Kyle et al., 2003; Cook et al., 2019; Facon et al., 2020; Cejalvo et al., 2021). Our study also showed similar results to those of previous studies.

Inflammation is an important hallmark of cancer that leads to the development and progression of malignancies. In established cancers, increasing evidence has shown that the progression of tumors and survival of patients with cancer are correlated with the local immune response and systemic inflammation (Diakos et al., 2014). Some studies have shown that the absolute NLR and MLR, as immune state indicators of patients with MM, are highly relevant to the survival of patients with newly diagnosed MM (Romano et al., 2015; Dosani et al., 2017; Romano et al., 2017; Binder et al., 2019). In the univariate analysis of OS in patients with EMM in our study, the NLR (≥4.2) and MLR (≥0.32) were associated with poor survival. In addition, in the LASSO Cox regression analysis, MLR remained a strong prognostic factor for OS.

The survival outcome of MM can be quite different due to its heterogeneity (Palumbo and Anderson, 2011); thus, no single staging system can ideally be applied to all patients with MM. Although the prognosis of EMM is worse than that of MM without extramedullary disease, establishing a nomogram predictive model is still necessary to predict individual survival, as the current staging systems of MM fail to provide enhanced accuracy and specificity in survival prediction in EMM. Moreover, with the development of new drugs, the number of regimens for EMM is increasing. Chimeric antigen receptor T-cell immunotherapy (CAR-T) has shown promising results in patients with EMM in several studies. In a first-in-human clinical trial of B-cell maturation antigen-targeted CAR-T therapy, Brudno et al. found that the therapy had encouraging activity against R/RMM. Among 16 patients, the ORR was 81%, with 10 of 16 (63%) achieving very good partial response or complete response. In addition, eradication of soft-tissue extramedullary plasmacytoma was also achieved (Brudno et al., 2018). A study enrolling 17 R/RMM cases treated with CAR-T also claimed that a high ORR of 88.2% was achieved, including 13 stringent complete response and 2 very good partial response. Satisfactory outcomes have also been observed in patients with EMM (Xu et al., 2019). Besides, central nervous system (CNS) involvement of EMM is an extremely rare (<1%), deemed as a very high-risk feature, always related with unfavorable cytogenetics, and, even with intense treatment applied, survival is usually less than 12 months. However, in our study, patients diagnosed of EMM had no central nervous system involvement. Matteo et al. reported the first patient with an extramedullary CNS relapse had response to targeted dabrafenib and trametinib treatment, providing evidence that a point mutation within the capicua transcriptional repressor (CIC) gene mediated the acquired resistance in this patient, which indicated that BRAF mutations might be a promising druggable target in multiple myeloma (Da Vià et al., 2020). Therefore, if high-risk patients can be identified by our model, they can receive more intensive treatments, such as CAR-T. Nomograms are widely used as prognostic tools in cancer and medicine. They can generate the individual probability of a clinical event by combining different prognostic factors, such as the individual probabilities of disease recurrence or death in patients. Compared with conventional staging, nomograms have increased accuracy, and prognoses are more easily understood, allowing clinicians to make rapid clinical decisions (Iasonos et al., 2008; Balachandran et al., 2015). To date, nomograms have become popular tools for predicting the clinical survival outcomes of various types of cancer (Liang et al., 2015; Tang et al., 2016; Ayubi and Safiri, 2017). Based on the prognostic factors we explored in patients with primary EMM in our study, we integrated powerful prognostic factors to build a nomogram to precisely predict survival in these patients. The discrimination and consistency of the predictive model were excellent compared with the current staging systems of MM.

However, our study had several limitations. First, this was a retrospective study conducted in a single center, and the number of patients was not large. Second, we could not collect adequate cytogenetic information to further explore its influence on EMM survival because most patients did not undergo cytogenetic examination. However, the cytogenetic features of EMD are not well-defined in literature. Third, we did not obtain an external dataset to validate our predictive model. Fourth, in our study, the ratio of the EMM patients received autotransplant was 10.6% in the whole cohort, which was low, so this may influence the validation ability of study. The reasons maybe as follows. First, the subjects enrolled in this study was from 2007 to 2021, which was a large time span. And fewer patients received autotransplant in the early years. Second, sometimes patients could not acquire enough stem cells for autotransplant. Third, some patients were unwilling to undergo autotransplant because of cost or other individual concerns. Therefore, the ratio of autotransplant in our study is lower than that of developed countries.

In conclusion, we developed a good predictive nomogram model based on the characteristics of EMM to predict individual survival probability with good discrimination and agreement. Large-scale and multicenter studies are warranted for further evaluation and validation.

## Data Availability

The raw data supporting the conclusions of this article will be made available by the authors, without undue reservation.
